# Schizophrenia: What We Know and What We Are Yet to Know

**DOI:** 10.7759/cureus.95890

**Published:** 2025-11-01

**Authors:** David Chinonyerem, Promise T Awe, Chisom O Okaro

**Affiliations:** 1 Health Sciences, University of Saskatchewan, Saskatoon, CAN; 2 Psychiatry and Behavioral Sciences, Walden University, Minneapolis, USA; 3 Psychiatry and Behavioral Sciences, Federal Medical Centre, Makurdi, Makurdi, NGA

**Keywords:** antipsychotic agents, genetics, mental health research, neurobiology, psychotic disorders, schizophrenia

## Abstract

Schizophrenia is an intricate mental disorder characterized by psychosis. The condition typically emerges during late adolescence and early adulthood, often persisting throughout a lifetime. The condition is understood to arise from a complex interplay of genetic, neurobiological, and environmental factors. This systematic review aimed to synthesize the extant knowledge state on schizophrenia through critical appraisal and integration of evidence from various domains, including neurobiology, genetics, clinical manifestations, therapeutic interventions, and environmental risk factors. We conducted an in-depth literature search on various medical databases, including PubMed, Medline, Scopus, and Google Scholar, for peer-reviewed articles focusing on schizophrenia. The study findings indicate that schizophrenia is a highly heterogeneous disorder attributable to complex interactions between neurobiological, genetic, and environmental factors, which make its diagnosis and treatment difficult. In our future studies, we intend to focus on interdisciplinary approaches and translation of molecular discoveries into clinical practice to improve diagnosis and treatment interventions, as well as reduce the schizophrenia burden.

## Introduction and background

Schizophrenia (Diagnostic and Statistical Manual of Mental Disorders, 5th Edition (DSM-5) code 295.90 (F20.9)) is a severe mental disorder affecting how individuals think, behave, and feel. Individuals with schizophrenia often experience psychosis with symptoms that range from delusions (strong, fixed, and false beliefs that are not founded on external realities and often persist regardless of clear evidence to the contrary) to hallucinations (false perception of an object or event, in any of the five senses, in the absence of an external stimulus) [1,2]. Individuals living with schizophrenia often lack a shared sense of reality and have been acknowledged to experience stigma as a result of behaviors striking other individuals as bizarre [1,2]. Mostly, persons with schizophrenia are highly likely to experience human rights violations within the community and mental health institutions’ contexts [3]. Often, stigma against individuals with schizophrenia is widespread and intense, resulting in social exclusion and affecting the individuals’ relations with others, such as friends and family members. This significantly contributes to discrimination that might also limit access to quality healthcare services, employment, education, and housing [3]. Additionally, individuals living with schizophrenia also struggle with confusing and scary experiences, and, as a result, often require understanding and compassion from those close to them, including family and friends. Consequently, schizophrenia has been linked to significant stress levels and impairments in family, personal, occupational, social, educational, and other vital spheres of life [4]. Individuals with schizophrenia are also three times more likely to die early compared to the general population [3]. This has been attributed to factors including physical illnesses such as metabolic, infectious, and cardiovascular diseases [3,4].

Significant efforts have been made in the recent past in a bid to demystify schizophrenia, with the initially prevailing psychoanalytic theories being discarded for increasingly robust and evidence-based models that acknowledge the condition as a brain function and structure disorder [5]. Further, advancements regarding what is known about schizophrenia included the established effectiveness of various antipsychotic medications that target dopaminergic pathways, and have been effective in the management of various positive symptoms [5,6]. Moreover, neuroimaging developments have disclosed significant and consistent anatomical considerations, including a reduction in the volume of gray matter and the enlargement of ventricular [7]. Further, various genetic studies have disclosed the existence of sturdy heritable components, acknowledging various risk alleles and concluding that no single gene is attributable to schizophrenia development [8]. On the other hand, existing biopsychosocial models have integrated different environmental risk factors, such as prenatal infections, urban upbringing, and childhood trauma, into an inclusive diathesis-stress model [1-4].

Regardless of such significant advancements, schizophrenia’s fundamental nature remains poorly understood, with several critical gaps persisting in our knowledge. Moreover, existing pharmacopeia, despite being life-changing for many individuals with schizophrenia, has failed to sufficiently tackle both cognitive and negative symptoms, considered the key determinants of long-term functional disabilities [9,10]. The exact pathophysiological mechanism that links genetic vulnerability and environmental elements to the development of schizophrenia remains unknown [9-11]. The increased heterogeneity in disease presentation and symptoms, disease course, and response to treatment is suggestive of schizophrenia not being a single disorder but a syndrome that comprises several distinctive etiopathological entities [11]. Despite being clinically important, the umbrella diagnostic term for schizophrenia also lacks the necessary biological accuracy needed for individualized medicine. What remains unknown about schizophrenia includes the search for effective biomarkers for early and prompt detection and prognosis, a deeper comprehension of the role of synaptic pruning and neuroinflammation, the development of newer therapeutic targets other than dopamine, and the execution of various effective interventions to enhance psychosocial outcomes while mitigating against stigma.

Although the extant literature on schizophrenia is vast, it is still fragmented, with the discoveries made in neuroscience genetics existing jointly with the persistent treatment challenges and several unanswered fundamental queries, including what the precise pathophysiological mechanisms of schizophrenia are, how the heterogeneity of schizophrenia can be resolved to enhance diagnosis and treatment, and how effective psychosocial interventions can be developed to enhance functional outcomes while reducing stigma. Therefore, the objective of this study is to effectively synthesize the existing knowledge state on schizophrenia through critical appraisal and integration of evidence from different domains, including neurobiology, clinical manifestations, genetics, therapeutic interventions, and environmental risk factors. Importantly, this study seeks to identify and subsequently delineate the existing gaps and contradictions in the literature, charting the vital course for future studies and clinical trials. Through systematically reviewing the known and unknown aspects of schizophrenia, this study aims to provide an increasingly comprehensive guide for researchers, policy makers, and clinicians for the discoveries required to alleviate the burden of schizophrenia.

## Review

Methodology

For this systematic review, an in-depth and comprehensive literature search was conducted on different electronic databases, including Scopus, PubMed, Web of Science, Google Scholar, and Embase for literature published between January 2015 and July 2025. The literature search strategy combined the identified MeSH keywords, including schizophrenia, psychotic disorders, neurobiology, genetics, antipsychotic agents, and mental health research, using wildcards and structured Boolean queries. Among the strings used in searching the databases included psychotic disorders[MeSH] OR schizophrenia[MeSH] AND genetics[MeSH] OR neurobiology[MeSH] AND antipsychotic agents[MeSH] OR mental health research [MeSH]. Consequently, wildcards were mainly applied in accounting for plurals and variations. Additionally, the searches were rerun before the final analysis to ensure inclusion of the most recent evidence. The studies selected for inclusion in this review included multicenter studies, prospective cohort studies, health assessment studies, systematic reviews, and epidemiological studies. The identification of duplicate data was done through a comparison of studies with comparable population years. The in-depth and comprehensive literature search performed resulted in a total of 38,840 references. A total of 18,691 duplicates were removed alongside 10,417 references marked ineligible by automation. Consequently, 9,732 references were screened, with 1,782 references being assessed for eligibility. Finally, eight studies met the inclusion criteria and were analyzed.

PICOS Framework

Population (P): As reported in peer-reviewed English-language articles from January 2015 to July 2025, the target population was individuals with a diagnosis of schizophrenia with no age, gender, or ethnicity limitation. Intervention/Exposure (I): The focus was on neurobiological, genetic, pharmacological, and environmental determinants of the onset, progression, and treatment of schizophrenia. Comparison (C): When relevant, comparisons were made between individuals with schizophrenia and those without, or between different subtypes of schizophrenia (e.g., resistant and responsive to treatment). Outcomes (O): The focus was on evidence regarding the causes and pathogenesis, symptom heterogeneity (positive, negative, cognitive), and treatment response or functional outcomes. Study design/Setting (S): The included studies were original quantitative research (e.g., cohort, cross-sectional, randomized, and quasi-experimental) and systematic reviews, with evidence from hospital, outpatient, and community settings qualifying.

PICOS question: Among individuals diagnosed with schizophrenia (P), how do neurobiological, genetic, pharmacological, and environmental factors (I), compared with healthy controls or across schizophrenia subgroups such as treatment-responsive versus treatment-resistant cases (C), influence symptom variability and treatment response (O) in peer-reviewed studies published between 2015 and 2025 (S)?

Inclusion and Exclusion Criteria

Following the removal of all duplicate studies, pertinent studies were further selected using a three-phase process. The first phase entailed the screening of the study titles and abstracts, whereas the second phase entailed the exclusion of inappropriate studies, including those with ineligible study designs, non-original research studies, studies whose population did not match the inclusion criteria, and studies with irrelevant interventions and exposure. The last phase entailed the performance of a complete full-text appraisal of all studies with the objective of verifying their significance. Three independent reviewers screened the references, and potential disagreements were mainly resolved using discussions and consensus.

The study inclusion criteria mainly targeted original studies, such as randomized controlled trials (RCTs), crossover studies, and prospective cohort studies that met the following set criteria: original study published in a peer-reviewed journal, study published between January 2015 and July 2025, study initially published in the English language, and full-text article. Additionally, a study had to focus on schizophrenia to be included. On the other hand, the exclusion criteria included opinion pieces, sponsored clinical trials, expert editorials, studies irrelevant to the target populations, and narrative reviews. Moreover, inappropriate and inaccessible studies, as well as studies with illogical methodologies, were excluded. Thus, the determination of *inappropriate studies* was mainly done based on the pre-defined exclusion criteria, alongside the inability to access the full text. Consequently, illogical methodologies were mainly identified during the performance of full-text review, given that the studies whose research design was unfitted to respond to their research questions. As a result, 348 studies were excluded.

Results

**Figure 1:**
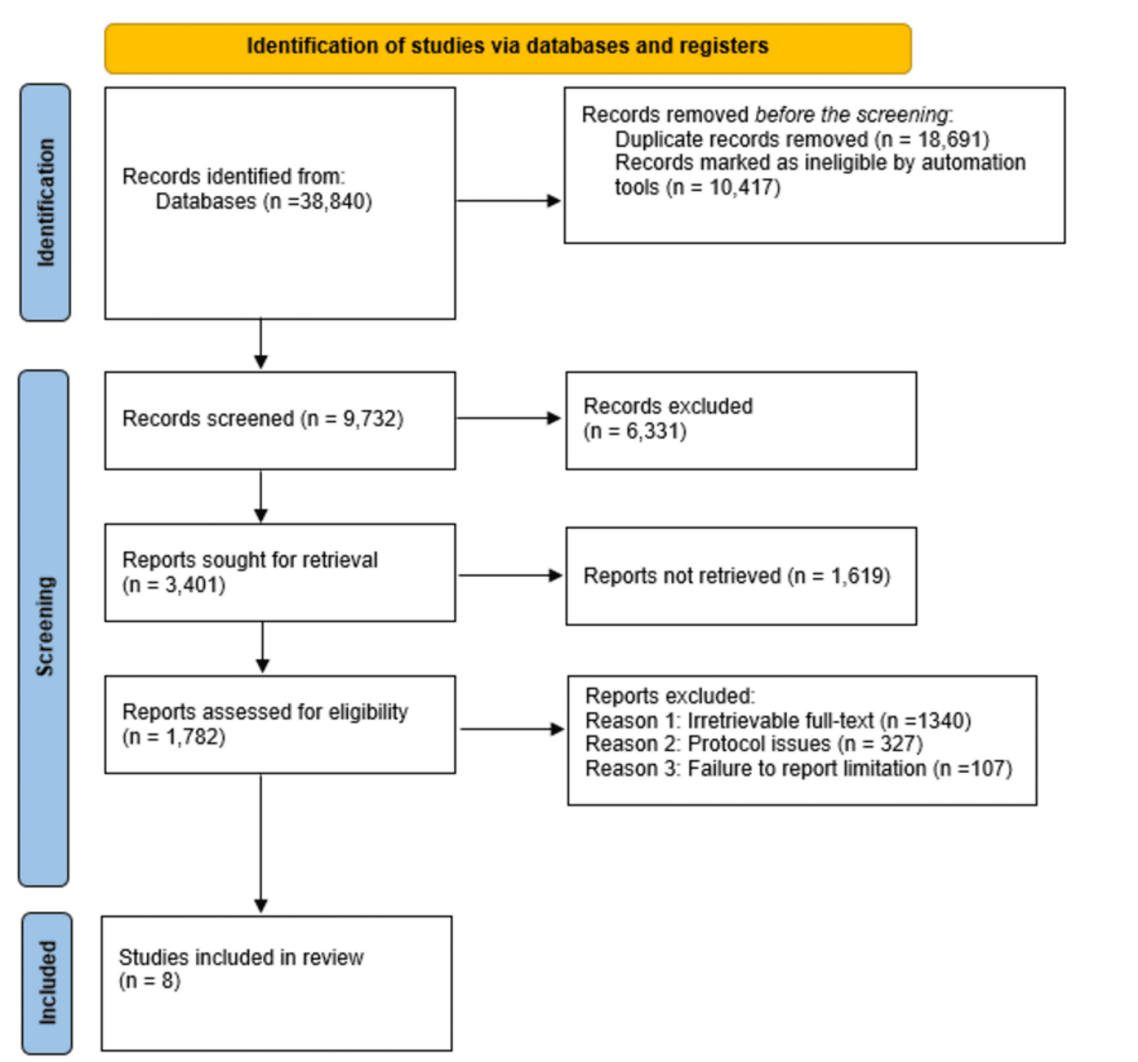
Preferred Reporting Items for Systematic Reviews and Meta-Analyses (PRISMA) flow diagram illustrating the study selection and inclusion criteria.

For this study, the Preferred Reporting Items for Systematic Reviews and Meta-Analyses (PRISMA) guidelines were used in the selection and inclusion of relevant literature. The first in-depth and comprehensive database search yielded 38,840 studies. However, after screening the studies, a total of 18,691 duplicates were excluded, while the succeeding screening of the titles and abstracts led to the automatic exclusion of 10,417 ineligible studies, for reasons such as inappropriate study populations, including inclusion of a mixed study sample in studies focusing on adults, inappropriate interventions and exposure, and inappropriate study designs. Of the remaining 9,732 studies sought for retrieval, 6,331 were excluded for reasons such as studies published in journals without available subscriptions and with paywalls without access, studies whose citations were incorrect and incomplete, and studies that were conference abstracts and theses without publicly available full texts. The remaining 3,401 studies were sought for retrieval, and 1,619 were found to be irretrievable, leading to their exclusion. As a result, 1,782 studies were assessed for eligibility, and 1,774 studies were further excluded for different reasons, including irretrievable full texts (1,340 studies), protocol issues (327 studies), and failure to report limitations (107 studies). Eventually, eight studies satisfied the inclusion criteria and were subsequently included. The included studies were reviewed and discussed together with the findings of other relevant studies, corroborating our findings and conclusions [3-36]. The PRISMA flow diagram outlining the article selection process for this study is shown in Figure 1.

Quality Assessment

All included studies’ quality was assessed using the AXIS Appraisal Tool for Cross-Sectional Studies, a 20-item critical appraisal tool for cross-sectional studies. Whereas three authors independently assessed each study, another two authors independently confirmed the assessments. Possible disagreements were solved through group discussions and consensus. Each study was subsequently accorded a score of 1 (yes), 0 (no), or “don’t know” for inapplicable items. Generally, the quality of included studies varied from moderate to high, with only two studies being rated moderate and the other six being rated high quality. The outcomes of the quality assessment conducted using the AXIS Appraisal Tool for Cross-sectional Studies are presented in Table 1.

**Table 1:** Outcomes of the quality assessment conducted using the AXIS Appraisal Tool for Cross-sectional Studies. N/A: not applicable

Authors, year	Clear objectives	Appropriate design	Target population defined	Sample size justified	Representative sample	Recruitment minimizes bias	Adequate response rate	Valid outcome measures	Blinding of assessors	Confounding addressed	Results support conclusions	Overall study limitations discussed	Ethical approval and consent	Conflicts of interest stated	Total "1" score/20	Overall quality impression
Lindhardt et al. [3]	1	1	1	0	1	1	1	1	N/A	1	1	1	1	1	16/20	High
Strauss et al. [8]	1	1	1	0	0	Don’t know	N/A	1	N/A	1	1	1	1	1	13/20	Moderate
Wainberg et al. [14]	1	1	1	1	1	1	N/A	1	N/A	1	1	1	1	1	18/20	High
Lewandowski et al. [19]	1	1	1	0	0	Don’t know	N/A	1	N/A	1	1	1	1	1	14/20	Moderate
Kegeles et al. [20]	1	1	1	0	0	Don’t know	N/A	1	N/A	1	1	1	1	1	14/20	Moderate
Millgate et al. [25]	1	1	1	0	0	Don’t know	N/A	1	N/A	1	1	1	1	1	14/20	Moderate
Chang et al. [35]	1	1	1	0	0	Don’t know	N/A	1	N/A	0	1	0	1	Don’t know	10/20	Low
Barrio et al. [36]	1	1	1	0	1	Don’t know	N/A	1	N/A	1	1	1	1	Don’t know	13/20	Moderate

Data Extraction

Three authors were tasked with extracting data, which was done independently. The data extracted from included studies comprised the study’s general attributes, including the sampling methods, author names, and year of publication; demographic attributes, including sample size, sex, age, race/ethnicity, and follow-ups; and data on interventions and durations. Each study’s major findings were systematically documented. Table 2 shows a summary of the findings of the studies included in this systematic review.

**Table 2:** Summary of the findings of the studies included in this systematic review.

Study/Citation	Primary focus	Key findings
Lindhardt et al. [3]	Prevalence of mental disorders in youth	A significant proportion (85.7%) of the individuals (youths) disconnected from education and work were reported to have undiagnosed schizophrenia and higher psychosis-risk syndrome rates (19.2%)
Strauss et al. [8]	The correlations between reinforcement learning, negative symptoms, and exploration in schizophrenia	The study disclosed that schizophrenia fractionates negative symptoms, with reduced expression associated with impaired reward learning and avolition linked to condensed exploration under uncertainty
Wainberg et al. [14]	The interaction between the use of cannabis and the genetic risk for schizophrenia in relation to psychotic experiences	Persons with a higher genetic risk for schizophrenia were found to be the most vulnerable to various psychosis-inducing effects related to cannabis use
Lewandowski et al. [19]	Cognitive variability patterns of psychotic disorders, including schizophrenia, schizoaffective, and bipolar with psychosis	The cluster analysis conducted disclosed the three transdiagnostic cognitive subgroups: cognitive impact, globally impaired, and selectively impaired, and cognitively intact, indicating that cognitive profiling was superior to clinical diagnosis for the psychosis patient subgrouping
Kegeles et al. [20]	The availability of dopamine D2/D3 receptors within the striatal and extrastriatal brain regions in persons with schizophrenia	The study utilized fallypride positron emission tomography imaging and disclosed that schizophrenia was linked to widespread dopamine dysfunction, as indicated by the significantly higher D2/D3 receptor availability throughout the striatal and extrastriatal brain regions in comparison to the healthy controls
Millgate et al. [25]	Analysis of cognitive variances between persons with treatment-responsive schizophrenia and those with treatment-resistant schizophrenia	Persons with treatment-resistant schizophrenia presented significantly greater cognitive impairment in all key domains compared to those with treatment-responsive schizophrenia, indicating that it might be an increasingly severe neurobiological form of the disease
Chang et al. [35]	Analysis of ethnic differences with regard to the symptom expression in severe schizophrenia	During acute admissions, African American schizophrenia patients reported significantly increased severe positive symptoms alongside excitement compared to their White American counterparts, indicating the significance of considering ethnic and cultural factors in clinical analysis
Barrio et al. [36]	Analysis of ethnic variances in symptom presentation on the Positive and Negative Syndrome Scale (PANSS)	The study disclosed that ethnicity had a significant influence on schizophrenia symptom presentation, with African Americans presenting increasingly severe positive symptoms while their Latino counterparts presented increased levels of emotional discomfort

Discussion

While schizophrenia has been considered a distinct disease entity over the years, its detailed definition and etiopathophysiology have remained obscure, even as its treatment has remained unsatisfactory [1]. In this regard, this systematic review has synthesized existing literature on the etiology, clinical presentation, pathophysiology, and management of schizophrenia. The review has disclosed that schizophrenia is an intricate and multifaceted disorder with an increasingly intricate interplay of factors, including neurobiological, environmental, and genetic factors.

Furthermore, despite having undergone revisions, the diagnostic criteria, an essential conceptualization, are still unchanged, as it is defined as a psychotic disorder with function/social impairment, with a propensity to be chronic, a psychosis onset during late adolescence and early adulthood, preceded by a prodrome [3,12]. In this regard, the International Classification of Diseases 11th Revision (ICD-11) and DSM-5 definitions require two specific symptoms, including a core psychotic symptom (disorganized thinking, hallucination, and delusion) [13]. Despite DSM integrating functional and social impairments as important parts of its definition, the ICD does not, even though it is a part of its schizophrenia description [13]. Additionally, the description of schizophrenia has been consistent over the years, despite the different aspects being accentuated at diverse times [12-15]. Initially defined as a protean disorder with increased variability across the illness course and patients [15], schizophrenia’s significant heterogeneity has been extensively highlighted in the last four decades [11]. However, the approach to clarifying and codifying such diversity has changed, as rather than subtypes (used between 1988 and 2008), symptom dimensions (multiple) are currently considered as effective in characterizing schizophrenia heterogeneity in ICD-11 and DSM-5 [16]. Still, alternative biological and psychopathological classification approaches have been developed in recent times [17].

Similarly, psychiatric symptoms of schizophrenia exist on a continuum from common symptoms to pathological symptoms, implying the diagnostic threshold of schizophrenia is still challenging. Thus, the clinical diagnosis of schizophrenia is increasingly reliant on the positive symptoms linked to prolonged psychotic episodes [14-19]. Nevertheless, approximately between 8% and 30% of the general population report having delusional experiences during their lifetimes, despite this being transient for most individuals [14-18]. Further, the psychotic symptoms of schizophrenia are not definitive of a given mental disorder [15,18,19]. The clinical efficacy of antipsychotic medications is increasingly related to their ability to effectively block the subcortical dopamine D2 receptors, indicating the significance of dopamine signaling [6,15-19].

Regardless of this, no consistent relationship has been found between schizophrenia pathophysiology and D2 receptors [20,21]. On the contrary, available clinical evidence has pointed to presynaptic dopamine dysfunction as the psychosis mediator in schizophrenia [20]. The comprehension of the neurobiology of schizophrenia continues to present significant challenges, even as one of the major features entails the subcortical dopaminergic dysfunction involvement in the various psychotic symptoms [22-25]. The existing knowledge on dopamine dysfunction has effectively clarified when and where dopaminergic alterations might exist in schizophrenia [20-23].

In this systematic review, the included studies collectively demonstrated a shift in the comprehension of dopaminergic alterations in schizophrenia [20-23]. In particular, persons with schizophrenia exhibited higher presynaptic dopamine function levels within the associative striatum in comparison to the limbic striatum. Additionally, such a pattern has also been found in individuals at high risk of schizophrenia [25]. As such, the perspectives on subcortical dopamine functions in schizophrenia have continued to evolve with the accommodation of such novel information. Nonetheless, basic studies conducted using animal models have gradually progressed in incorporating clinical findings. For instance, the widely employed phenotype for positive schizophrenia symptoms in rodent models, psychostimulant-induced locomotion, has been linked to dopaminergic activation within the limbic striatum [23-26]. Such anatomical misalignments have led to queries regarding the best approach to assessing positive symptoms using animal models, and subsequently represent an essential opportunity for enhanced translation between basic and clinical research.

In the last four decades, neurobiological studies focusing on schizophrenia, supported by advanced imaging, have corroborated and expanded on the initial findings [22]. Among the notable structural changes associated with schizophrenia are the enlargement of the lateral ventricles, reduction in brain volume, and a significant loss of grey matter in the frontal lobes and hippocampus [22]. Schizophrenia has additionally been marked by extensive white matter abnormalities alongside impaired connectivity between the brain networks. Still, anomalies of the neurotransmitter system have been acknowledged, including the mesolimbic dopaminergic hyperfunction, which underlies GABA and glutamate imbalance and psychosis [26]. The immune aberrations and episodic inflammatory processes have been widely observed in schizophrenia [27]. Such diverse biological abnormalities have been directly associated with cognitive, positive, and negative symptoms defining schizophrenia. Comparable neurological alterations, including changes in functional connectivity and cortical thinning, have been reported in other psychiatric syndromes such as major depression and bipolar disorder, which are liable to complicate the differential diagnosis.

Significant efforts have been made to find the correlation between genes and the environment in the development of schizophrenia. Thus, even as the interactions between genes and the environment may be of immense significance in modifying the risk and expression of schizophrenia, the process of accurately elucidating their nature remains in its infancy [28,29]. Recent cadaveric studies focusing on the prefrontal cortex with samples drawn from hundreds of postmortem brains of schizophrenia and normal subjects have disclosed that the cortical GABAergic and glutamatergic systems are significantly affected by modest changes in histone acetylation, and, more significantly, the chromatin function and structure, at several hundred genomic loci [26]. Furthermore, the sequences impacted by such and other key epigenomic alteration types, such as DNA cytosine methylation, have shown substantial enrichment for schizophrenia genome-wide association study loci, demonstrating that epigenetic changes within the brains of individuals diagnosed with schizophrenia track the disease’s genetic risk architecture [26,30]. Still, several studies have revealed that the occurrence of epigenetic dysregulation at the schizophrenia risk loci site involves several highly expressed or regulated genes during fetal brain development [26,31].

The other aspect of schizophrenia that remains unknown is whether the same genes are responsible for the mental disorder in different populations. Genome-wide experimental studies are rapidly altering the comprehension of schizophrenia’s molecular genetics. Such studies have also disclosed infrequent copy number variations, mostly deletions, linked to schizophrenia and the common single-nucleotide polymorphism with schizophrenia-associated alleles [32]. Aggregate data have significantly supported the theory of polygenic inheritance alongside genetic overlap of schizophrenia with bipolar and autism disorders [33]. Nevertheless, schizophrenia belongs to the complex genetic disorders group of pathologies. The existing knowledge of complex genetic disorders is continually evolving as novel experimental studies reveal new disease mechanisms. It is widely believed that several genes are actively involved in every mental disorder, with each conferring only a reduced effect on the phenotype [31,32]. Therefore, the individual risk variants do not have adequate predictive power for diagnostic purposes, even though existing and up-to-date risk approximations are anticipated to be more refined through the prospective analysis of larger epidemiological cohorts [33,34]. The epistatic interactions that occur between such genes, as well as between their products, and the interactions that occur with various environmental risk factors are plausible. Even so, the study of gene interactions using genome-wide data is largely unexplored, given the need to correct for a significant number of statistical comparisons. Further, schizophrenia knowledge is also shifting from the original oligogenic models toward a more polygenic model; however, the genetic architecture of schizophrenia is still largely misunderstood. Existing evidence strongly suggests that the mutation frequency spectrum encompasses a combination of rare and common mutations [30]. The notion that complex mental disorders are not attributable to the abnormal function of an individual gene, but rather as a result of the entire molecular network dysfunction, the system disorder theory, has been highlighted in recent studies [35]. It is not yet understood whether this applies to schizophrenia.

While it is already known that schizophrenia is a mental disorder that comprises numerous distinct illnesses, it is noteworthy that what leads to certain symptoms being increasingly expressed in a patient and a divergent subset in another patient remains unknown. In this regard, various studies have been conducted to elucidate the differences in symptom expression. For instance, a recent study by Chang et al. disclosed the existence of broader variations in symptom presentation among schizophrenia patients from dissimilar ethnic backgrounds in the course of a schizophrenia episode [36]. Various studies have disclosed that African American patients presented an increased number of positive and negative symptoms, even as other studies disclosed that Euro-American schizophrenia patients expressed an increased number of positive and negative symptoms compared to the Latino and African American patients [30,35,36]. Such a heterogeneity of study findings shows the diversity of symptom expression across racial and ethnic groups in persons with schizophrenia. The heterogeneity of such findings accentuates not only the symptom expression diversity but also the important cultural divergences influencing the behaviors that are focused upon and subsequently pathologized. The contradictory results might arise from cultural differences in the way patients express distress, as well as from clinician bias with regard to the interpretation of such behaviors through a primarily Western diagnostic lens. Consequently, such disparities underscore the need for culturally sensitive evaluation tools to differentiate genuine clinical symptoms from culturally normative behaviors. Despite significant progress in research on neurobiological sciences and pharmacology, precise prognostic markers for accurate illness forecasting remain nonexistent [9-11]. Recognition of the condition and subsequent intervention still depend on clinical symptom presentation, which, in most cases, leads to an untimely diagnosis and treatment intervention. This, in turn, diminishes the chances for timely psychiatric intervention [6,19,20].

Future research should focus on the creation of neuroimaging and genetic epigenetic integrative biomarker panels, as their validation in large longitudinal cohorts would allow for earlier diagnosis and enable more precise prognostic stratification. The prescribed treatments for schizophrenia center on antipsychotic medications. These treatments primarily target dopamine receptors and, while they can alleviate positive symptoms, they are ineffective in treating negative symptoms and cognitive deficits [6,9,10]. Despite the absence of treatment options, these domains are pivotal when determining the functional outcomes and long-term disability of an individual suffering from schizophrenia [6,9,10,18,19]. Further, future research should focus on devising alterations for therapeutic approaches that do not center on dopamine antagonism and instead utilize glutamatergic, GABAergic, and inflammatory pathways in combination with cognitive remediation. The symptomatology of schizophrenia reveals profound clinical diversity as the symptoms differ across varying ethnic and cultural groups [36]. Certain populations experience positive and negative symptoms of greater severity, but psychotic symptoms also occur momentarily in the general population [18]. This diversity is challenging to categorize and diagnose because no single model exists to account for these differences [11,18,36]. Additional studies should employ cross-ethnic research designs to develop more integrative and biologically accurate classification systems that account for cultural diversity and disease variability.

## Conclusions

In summary, this study has disclosed that the existing knowledge of schizophrenia remains deep but narrow. There is a sturdy, albeit incomplete, understanding of schizophrenia’s neurodevelopmental and genetic origins, as well as a reliable but limited treatment alternative for the positive symptoms. Moreover, even as the advancements in genetics, imaging science, and neurobiology have offered vital insights into epigenetic regulation, structural brain abnormalities, and dopamine dysfunction, no explanatory model has been developed to account for schizophrenia’s divergent clinical expressions. The observed persistence of uncertainties in diagnosis alongside the cross-cultural variability in the expression of symptoms has also emphasized the need for additional integrative models capable of bridging the biological, environmental, and genetic perspectives. Therefore, in our future prospective studies, we intend to focus on interdisciplinary approaches and translation of molecular discoveries into clinical practice to improve diagnosis and treatment interventions, as well as reduce the schizophrenia burden.
